# Comparing the Efficacy of Formaldehyde with Hydrogen Peroxide Fumigation on Infectious Bronchitis Virus

**DOI:** 10.1177/1535676020909998

**Published:** 2020-04-15

**Authors:** Jamie Stuart, John Chewins, Jason Tearle

**Affiliations:** 1 The Pirbright Institute, Pirbright, Surrey, UK; 2 Bioquell UK Limited, Andover, Hampshire, UK

**Keywords:** fumigation, hydrogen peroxide, infectious bronchitis virus, formaldehyde, coronaviruses

## Abstract

**Background:**

The recent reclassification of formaldehyde as a presumed carcinogen prompted the investigation into the comparative efficacy of hydrogen peroxide as a fumigant in microbiological safety cabinets.

**Introduction:**

The aim of the study was to quantify the biocidal efficacy of formaldehyde fumigation, including variables such as exposure time and concentration, and then to compare this to the biocidal efficacy achieved from a hydrogen peroxide vapor fumigation system. The study also investigated the ability of both fumigants to permeate the microbiological safety cabinet (MBSC), including the workspace, under the work tray, and after the HEPA filters. Furthermore, the effect of organic soiling on efficacy was also assessed. Infectious bronchitis virus (IBV) was used as the biological target to develop this study model.

**Methods:**

A model using IBV was developed to determine the efficacy of formaldehyde and hydrogen peroxide as fumigants. Virus was dried on stainless steel discs, and variables including concentration, time, protein soiling, and location within an MBSC were assessed.

**Results:**

It was demonstrated that formaldehyde fumigation could achieve a 6-log reduction in the titer of the virus throughout the cabinet, and high protein soiling in the presentation did not affect efficacy. Appropriate cycle parameters for the hydrogen peroxide system were developed, and when challenged with IBV, it was shown that vaporized hydrogen peroxide could achieve an equal 6-log titer reduction as formaldehyde within the cabinet workspace and overcome the presence of soiling.

**Conclusion:**

Hydrogen peroxide was demonstrated to be a viable alternative to formaldehyde under most situations tested. However, the hydrogen peroxide system did not achieve an equal titer reduction above the cabinet’s first HEPA filter using the cabinet workspace cycle, and further optimization of the hydrogen peroxide cycle parameters, including pulsing of the cabinet fans, may be required to achieve this.

## Introduction

Formaldehyde, the simplest aldehyde, is an organic compound also known as methanal that has the molecular formula CH_2_O, commonly shown as HCHO. When dissolved in H_2_O, often with methanol as a stabilizer, the solution is known as formalin. Since the start of the 20th century, it has been known that vaporized formalin can be used as a disinfectant of microbiological organisms,^1^ and currently there is commonplace use of formaldehyde as a chemical fumigant in medical, laboratory, and pharmaceutical environments to microbiologically decontaminate. Formaldehyde fumigation is a popular fumigation method because it is easy to set up, relatively cheap, and well established as a dependable method for achieving decontamination.

The main disadvantage of formaldehyde is its likely carcinogenic properties. In January 2016, the European Union (EU) officially adopted the reclassification of formaldehyde (CLP Regulations EC 1272/2008) as a Class 1B carcinogen (ie, a presumed human carcinogen) and Class 2 mutagen. This prompted the United Kingdom’s Health and Safety Executive (HSE) to recommend that all users of formaldehyde in a laboratory decontamination setting investigate alternative gaseous disinfectants while the use of formaldehyde is under review. The use of an alternative hydrogen peroxide fumigation system was then investigated to validate its efficacy against infectious bronchitis virus (IBV).

Hydrogen peroxide has several advantages over formaldehyde, primarily that the vapor is less hazardous to human health. It breaks down into water and oxygen, meaning it requires no postprocess neutralization and leaves no residue. When vented out of the air handling system, this also makes hydrogen peroxide more environmentally friendly than formaldehyde, which breaks down to form carbon monoxide and formic acids, components of acid rain. However, relative to formaldehyde, the hydrogen peroxide systems are currently far more expensive. In addition, when compared to the simplicity of formaldehyde, different situations may require bespoke hydrogen peroxide cycle parameter setups.

Formaldehyde acts as a biocide by attacking the primary amide and amino groups of peptides,^2^ therefore forming intermolecular methylene bridges between proteins^3^ as well as by alkylating the nitrogen atoms of nucleotide bases in DNA and RNA. By comparison, hydrogen peroxide (H_2_O_2_) is an oxidative biocide that may generate radicals to broadly oxidize biomolecules across the target,^4^ such as membrane proteins, enzymes, or nucleic acids.^5^ Both modes of action can cause organic damage to lead to cell death or virus inactivation.

There is a growing resource of data currently available on the efficacy of various hydrogen peroxide fumigation systems in specific settings. Within sealed rooms or isolators, hydrogen peroxide has been shown to be capable of causing complete decontamination of certain bacteria,^6^ bacterial spores,^7,8^ fungal spores^9^ and viruses.^10,11^ For studies of fumigation efficacy, there is common use of sporulating bacteria such as *Geobacillus stearothermophilus* and *Bacillus atrophaeus* to validate the fumigation process due to the known high resistance of these endospores to environmental degradation.^12^ The assumption that tends to be made is that full reduction of these endospore populations by fumigation therefore validates the decontamination process against all other potential biological targets. However, it has been shown that commercially available bacterial spore indicator strips do not always reflect the inactivating capacity of a fumigant against other agents, particularly viruses.^13^ Consideration should therefore be given to conducting the initial validation of fumigation processes using the actual target organism. In addition, it should be noted that the studies published on the efficacy of hydrogen peroxide often use different types of H_2_O_2_ systems, different-sized target areas, and different target organisms. This makes it difficult to truly validate a new fumigation process against a specific target without prior testing of the system in situ.

To validate the use of a hydrogen peroxide fumigation system, there first needs to be an established baseline for the efficacy of the current formaldehyde fumigation procedure. Due to the long historical use of formaldehyde, much is known about the optimal method of use. It is important that alongside the solution of formalin, there is an adequate volume of water so that, when vaporized, a sufficiently high level of relative humidity can be achieved within the fumigated area, which is essential for successful decontamination.^14^ There are several examples of using this process to successfully inactivate a range of organisms, from bacterial spores^15,16^ to viruses,^17,18^ including IBV^19^ used in this study.

The current European standard for biosafety cabinets (BS EN 12469:2000) describes a method of using liquid formalin and water at a ratio of 60 mL/60 mL per cubic meter (m^3^) of cabinet volume. However, it has been noted that there is no obvious reference provided on the development and validation of these volumes.^20^ It is apparent that different institutions use their own ratios, and work undertaken at HSL (Health & Safety Laboratory) has shown that this standard cabinet ratio may be higher than required and does not necessarily convert to larger room volumes.^21^

The objectives of this study were to quantify the biocidal efficacy of formaldehyde fumigation and then to compare this to the biocidal efficacy achievable from a hydrogen peroxide fumigation system. The work was done using IBV, a highly contagious virus causing economically important viral disease in chickens, as the biological target to represent a commonly used enveloped virus that the fumigation procedures would be expected to decontaminate.

## Methods

### Presenting Target Virus

For each fumigation experiment, IBV (strain Beau-R) was presented in a controlled way within a Class II microbiological safety cabinet. Stainless steel discs 2 cm in diameter were placed centrally within the cabinet workspace. The discs were provided by a microbiological safety cabinet (MBSC) manufacturer (Walkers Safety Cabinets, Glossop, UK) and were intended to represent the material virus may be deposited and dried on within an MBSC. The discs were cleaned and autoclaved after each use. Then, 100 μL of IBV stock solution of known titer was dispensed centrally onto the surface of each disc. This was left to visibly dry for approximately 2 hours with the cabinet left on. Once dried, a positive control disc was removed from the cabinet and the others subjected to the fumigation procedure.

### Recovering and Quantifying the Virus

To recover the remaining virus from each disc, the method used was adapted from existing methods.^22^ Then, 100 μL of phosphate-buffered saline (PBS) solution was pipetted directly onto the surface of the disc where the virus had originally been deposited. The same 100 μL of PBS was then forcefully pipetted up and down on this position 30 times to wash all virus particles off the surface and into solution. The 100-μL wash solution was then transferred into 900-μL 1× BES (1×E-MEM, 0.3% tryptose phosphate broth, 0.2% BSA, 20 mM N,N-Bis(2-hydroxyethyl)-2-aminoethanesufonic acid (BES), 0.21% sodium bicarbonate, 2 mM L-glutamine, 250 U/ml nystatin, 100 U/ml penicillin and 100 U/ml streptomycin) cell culture medium, which was taken forward to be used as the first 10^–1^ dilution for the plaque assay. The plaque assay was carried out as previously described by Baer and Kehn-Hall^23^ with solid agar overlays but using primary chicken kidneys cells (CKCs) and BES cell culture media. Each data point represents the average titer reduction of 3 discs.

### Organic Contamination of Virus Samples

To simulate high levels of protein soiling around the biological target, some of the experiments used discs inoculated with IBV as well as fetal bovine serum (FBS). For these samples, the same process as before was applied except the 100-μL IBV stock solution was mixed with 100 μL FBS prior to being deposited on the disc and the drying time increased to 3 hours. During recovery, the deposition site was first aggravated with a pipette tip to dislodge the fixed protein layer; the same recovery procedure as before then applied, after which a sterile swab was used to transfer any remaining clumps of FBS into the recovery solution where the swab tip was cut off and left in the solution.

### Formaldehyde Fumigation

The desired volume of formalin (39% w/v) was deposited into a formalin vaporizer placed within the cabinet. A volume of H_2_O was added according to the following formula:

$${\text{H}}_{2}\text{O}\left(\text{mL}\right)\;=\;10-(\text{HCHO}\left(\text{mL}\right)/2),$$

where H_2_O(mL) is the volume of water to be added and HCHO(mL) is the volume of formalin already added. This allowed the variable of relative humidity to be controlled for each experiment. Note that this formula did not apply to the standard 20-mL/20-mL mix used routinely for MBSC fumigation. Once the cabinet was sealed, the mixture was vaporized and allowed to dwell overnight for ∼18 hours, the exception being the 1-hour shortened dwell study. Aeration was done using the cabinet extract system to purge the air into the air handling ventilation system for ∼1 hour.

The volume of the 1200-mm cabinet used for fumigation was approximated at 333 L, and the standard mixture used was 20 mL formalin and 20 mL water, which is in line with the current European standard for biosafety cabinets (BS EN 12469:2000). Depending on the volume of formalin (39% w/v) used, the concentration of vaporized formaldehyde in the 333-L cabinet was calculated as a value in parts per million (ppm), and the concentration was not directly measured. For example, the 20-mL formalin standard mixture can here be approximated to 18 000 ppm.

### Hydrogen Peroxide Fumigation

A Bioquell (Andover, Hants, UK) Clarus C hydrogen peroxide vapor (HPV) generator was the system used for all fumigation experiments. Bioquell HPV-AQ hydrogen peroxide solution (35% w/w) was used as the biocidal agent. Two different cycles were programmed and used in this study (Table 1).

**Table 1.** Hydrogen Peroxide Cycle Parameters.

Cycle Stage	Cabinet Workspace (Cycle 1)	HEPA Cycle (Cycle 2)
Conditioning	10 min	10 min
Pregassing	1 min	1 min
Gassing	15 min @ 3 g/min H_2_O_2_	35 min @ 3 g/min H_2_O_2_
Dwell	30 min @ 0.5 g/min H_2_O_2_	30 min @ 0.5 g/min H_2_O_2_
Aeration	90	90

The aeration was completed by first using the Bioquell Clarus C systems internal catalyst to promote the H_2_O_2_ breakdown for 30 minutes before using the cabinet extract system to purge the air into the air handling ventilation system for ∼1 hour.

All statistical analysis was performed using GraphPad Prism v7 (GraphPad Software, La Jolla, California). For the stated *P* values, the statistical significance was determined by 1-way analysis of variance (ANOVA) with post hoc Tukey honestly significant difference (HSD) test.

## Results

To validate all subsequent experiments fumigating live virus, the method developed for recovering the dried virus samples (as described in Methods) must be shown to be reliable. Three repeats showed ∼1-log reduction in virus titer after the drying and recovery process. There was no statistically significant difference between the average titer reduction of any of the repeats. This shows that the process could consistently recover the same concentration of virus, and therefore, in subsequent experiments, any changes in titer seen postfumigation can be attributed to the fumigation procedure itself and not the virus recovery method.

To establish an initial baseline for the efficacy of formaldehyde fumigation against IBV, the same cabinet was subjected to fumigation with formaldehyde at a range of concentrations. At formaldehyde concentrations less than ∼700 ppm, there starts to be virus survival within the cabinet after fumigation, which steadily increased as the formaldehyde concentration decreased. To further test the efficacy of formaldehyde fumigation, an experiment was carried out to determine the potential biocidal effect beyond the cabinet workspace. Target discs of dried IBV solution were positioned internally within the cabinet, below the cabinet workspace, and above the first extract HEPA filter. The experiment was repeated 3 times, each run with 3 discs. At formaldehyde concentrations of 18 000 ppm, there was still a full 6-log virus titer reduction below the workspace that was mirrored above the first HEPA filter. By comparison, at 900 ppm, there was a significant difference both above the first HEPA filter (*P* = .0036) and below the workspace (*P* = .0207). At 900 ppm below the workspace, the average titer reduction was still greater than a 4-log reduction, although above the first HEPA filter, the average titer reduction was less than 4 logs (Table 2).

**Table 2.** Summary of Virus Inactivation (Log_10_) for Formaldehyde and Hydrogen Peroxide at Varying Concentrations, Cycle Parameters, and Locations Within the MBSC.

	Formaldehyde (ppm)		H_2_O_2_ Cycles (see Table 1)		
Characteristic	18 000	900	Workspace Decontamination (Cycle 1)	Workspace Decontamination (Cycle 1) with Fans Pulsing	HEPA Cycle (Cycle 2)
MBSC work area	6	6	6	6	6
MBSC under work tray	6	5	6	6	6
MBSC postextract HEPA	6	3	<2	3	2

Abbreviation: MBSC, microbiological safety cabinet.

^a^ All numbers are rounded log_10_ infectious bronchitis virus titer reduction values.

A challenge that fumigants often face is that the biological target is not always cleanly presented; often, there is considerable organic or proteinaceous soiling. To simulate a high level of protein contamination, the target samples were supplemented with equal volumes of FBS. It is hypothesized that during formaldehyde fumigation, the protein in the sample will fixate to create a shielding layer potentially offering protection to virus particles within.

It was observed that above a concentration of ∼1140 ppm, there was no difference in the average titer reduction between virus samples with and without added FBS.

**Figure 1.**
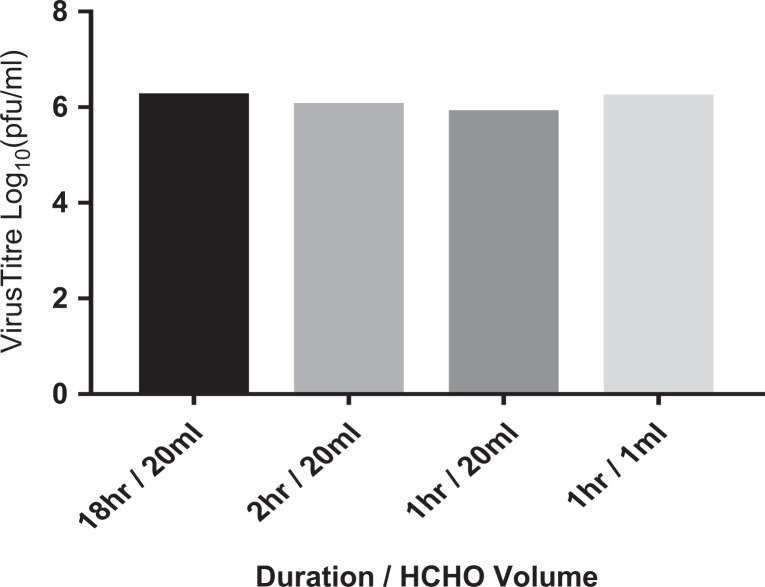
Effect of dwell duration on virus titer reduction. Virus titer is the average titer reduction postrecovery compared to a dried positive control.

All previous experiments on formaldehyde fumigation had used a dwell time of ∼18 hours. The dwell is the length of time that the vaporized solution of formaldehyde and water is left within the cabinet before aeration starts. To better understand the biocidal efficacy of formaldehyde, fumigations with reduced dwell time were carried out. An overview of the findings is shown in Figure 1, with each bar representing the average titer reduction of 3 discs. Using the standard ratio of 20 mL formaldehyde and water, the dwell time was reduced to 2 hours, then 1 hour, long and there was still a complete reduction in virus titer as seen before with the 18-hour dwell time. The volume of formaldehyde added to the vaporizer was then reduced to 1 mL (cabinet concentration of ∼900 ppm) for 1 hour. Using 1 mL for 1 hour, it was found that there was still no change in the average titer reduction (ie, complete reduction).

**Figure 2.**
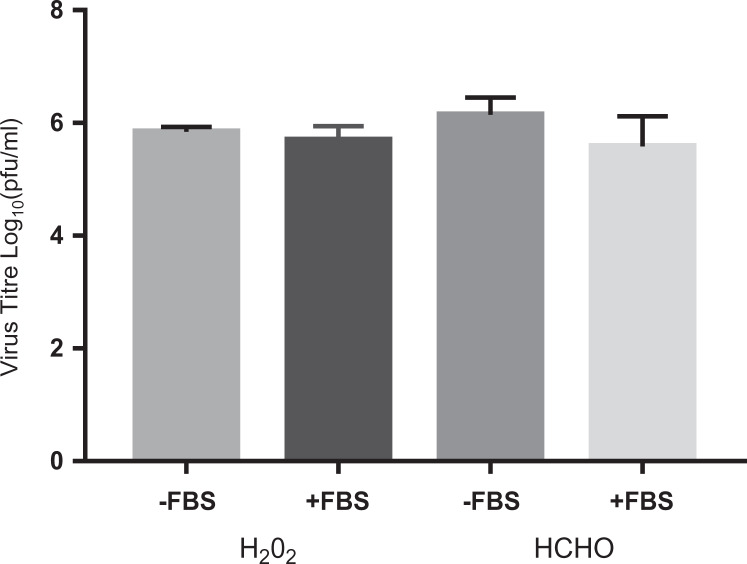
Comparison between H_2_O_2_ and HCOH fumigation both with and without fetal bovine serum (FBS) added. H_2_O_2_ “normal” virus cycle used. [HCOH] at ∼18 000 ppm. Virus titer is the average titer reduction postrecovery compared to a dried positive control.

Fumigation using the hydrogen peroxide vapor system was done under the same conditions as were used for formaldehyde fumigation. The same Class II MBSC was used and the IBV samples were presented in an identical way. A hydrogen peroxide cycle was developed and programmed with guidance from the manufacturer to try and optimize for the cabinet size and biological target. Figure 2 shows the results of IBV samples subjected to hydrogen peroxide fumigation within the cabinet workspace. Each bar is the average of 3 runs, where each run is the average of 3 discs. For comparative purposes, the results of standard ratio formaldehyde fumigation (∼18 000 ppm) are shown alongside. Using the hydrogen peroxide system, an average titer reduction of 6 logs was achieved, which is equivalent to that achieved through formaldehyde fumigation. With addition of FBS to the IBV samples to simulate high levels of protein soiling, the hydrogen peroxide system was still capable of achieving a full 6-log reduction in virus titer. There was no statistically significant difference between the average titer reduction achieved using either formaldehyde or hydrogen peroxide fumigation.

The hydrogen peroxide fumigation system was then challenged further by placing the virus samples at the internal cabinet locations previously tested with formaldehyde—both below the cabinet workspace and above the first HEPA filter. Initially, the hydrogen peroxide system was run with the same fumigation cycle parameters as those designed to biodecontaminate just the cabinet workspace. Below the workspace, a full 6-log reduction in the average titer was achieved. However, above the first HEPA filter, the average titer reduction was less than 2 logs, a significant difference in virus survival compared to the results from formaldehyde fumigation at the same location (*P* < .001) (Table 2).

It was noted that the formaldehyde fumigation method may have had an unfair advantage in this test of penetrative ability due to the cabinet being placed in a “fumigation mode” where the fans pulsed for 5 seconds at 20-minute intervals for the first hour to aid fumigant circulation. Therefore, the experiment was repeated but with the cabinet fans pulsing during the hydrogen peroxide fumigation process as well. This increased the average titer reduction achieved above the first HEPA filter using hydrogen peroxide to over 3 logs. This is, however, still significantly lower (*P* = .0003) than the average titer reduction that formaldehyde fumigation was capable of. This experiment was conducted only once as it was considered more useful to extend the gassing phase to account for the greater surface area and absorbency of the HEPA filter media. The experiment was repeated with the hydrogen peroxide system using the new cycle parameters, but the cabinet was not placed in fumigation mode (ie, the fans were not pulsed). The extended cycle did not significantly increase the biocidal efficacy of the system as the average titer reduction remained at around 2 logs (Table 2).

## Discussion

Once the methodology had been validated, the first aim of the study was to quantify the efficacy of formaldehyde fumigation using IBV as the biological target. Fumigating over a range of concentrations revealed that against IBV, there was a complete reduction in virus titer down to a formaldehyde concentration of ∼700 ppm. The threshold of fumigation failure around this concentration is considerably lower than the concentration of ∼18 000 ppm currently used to fumigate cabinets. The volume of formaldehyde currently used could be significantly reduced, and large reductions in virus titer within the cabinet workspace would still be achieved.

When formaldehyde was tested against IBV samples located at internal cabinet locations beyond the workspace, the fumigant demonstrated proficiency at penetrating into the cabinet. At the currently used standard concentration of ∼18 000 ppm, the formaldehyde fumigation achieved full 6-log titer reduction below the workspace with nonsignificant virus survival above the first HEPA filter. Even at the considerably lower formaldehyde concentration of ∼900 ppm, there was a high average titer reduction at the internal cabinet locations of approximately 5 logs below the work tray and 3 logs above the extract HEPA filter. The results at ∼900 ppm showed that the position above the first HEPA filter proved the most challenging location to achieve a biocidal effect. This was not unexpected seeing as the fumigant would have to travel the furthest to reach this location, and the HEPA filter itself might trap fumigant passing through.

It has been previously observed that high levels of organic contamination around the biological target can make it harder to achieve the desired biocidal effect,^24^ which has been hypothesized to be due to the formation of a surface barrier of fixed protein potentially hindering the deeper penetration of formaldehyde,^17^ therefore shielding the virus. This study found that organic soiling simulated by a high level of FBS protein around the biological target did result in a protective effect on the virus sample, but it should be noted at low formaldehyde concentrations, the effect was less. Even so, the concentration of proteinaceous soiling in this study did not influence fumigation efficacy using the current standard concentration of formaldehyde used when fumigating MBSCs. Overall, this demonstrates that formaldehyde fumigation can cope well with organic soiling but still promotes the concept of wiping down the target area prior to fumigation commencing.

Interestingly, the initial data from this study suggest that the standard 18-hour overnight dwell time currently used for formaldehyde fumigation is much longer than may be required for the biocidal action to take place, particularly within the workspace of the cabinet. It was found that the reduction in virus titer was the same for both a 1-hour dwell time and an 18-hour dwell time on indicators placed in the workspace, meaning that the full biocidal effect was happening within the first hour of the fumigation process. Similar observations have been made using commercial indicators containing a surrogate organism (author’s personal observation). Although overnight fumigation may still be convenient, these data suggest that it may not be necessary.

The hydrogen peroxide system was tested against IBV in the same cabinet as the formaldehyde fumigation to generate comparable results to determine if the hydrogen peroxide system could achieve the same biocidal efficacy as demonstrated by formaldehyde at the currently used standard concentration. In the cabinet workspace, hydrogen peroxide fumigation was able to achieve a 6-log average titer reduction, equivalent to that of formaldehyde. Previously, it has been suggested that organic soiling could reduce the efficacy of hydrogen peroxide fumigation,^7,9^ but in this study, a full 6-log average titer reduction was still achieved when the FBS soiling was present around the IBV sample. It is worth noting that soiling in the form of blood has been argued to have a more pronounced effect^10^ due to the presence of peroxidase and catalase enzymes that could neutralize the hydrogen peroxide. Further testing of the hydrogen peroxide system against organic soiling could use blood or related tissues as an alternative challenge.

When the hydrogen peroxide system was also challenged with IBV samples at internal cabinet locations, the fumigant was able to achieve full titer reduction below the workspace, but above the first HEPA filter, there was a significantly lower average titer reduction compared to the results for formaldehyde using the 20-mL/18 000-ppm process (*P* < .001). This was found to be the case with the cabinet workspace biodecontamination cycle parameters as well as when the length of the gassing phase was increased. To attempt to promote circulation of the vaporized hydrogen peroxide around the cabinet, the experiment was repeated with the cabinet turned on in “formaldehyde fumigation mode” where the fans pulse periodically (5 seconds at 20-minute intervals for the first hour), using the cabinet workspace biodecontamination cycle parameters. Although this did improve the average titer reduction achieved, it was still too low and would be classed as fumigation failure. Running the extended “HEPA” cycle in conjunction with pulsing, the fans may have achieved a greater reduction. From this study, it can be concluded that the hydrogen peroxide system using the cabinet workspace cycle parameters was unable to achieve adequate biocidal effect above the HEPA filter. Extending the cycle parameters without pulsing the fans also did not achieve the required log reductions. Making direct comparisons to the results achieved with formaldehyde must take into consideration the differences in dwell time. The hydrogen peroxide cycles used in this study had dwell times of 30 minutes, whereas the formaldehyde cycles dwelled for 18 hours. One advantage of hydrogen peroxide cycles is that cycle times are usually much shorter, allowing quicker turnaround and less impact on laboratory activities. Further optimization of the cycle parameters, including appropriate pulsing of the fans to drive the hydrogen peroxide through the filter, may allow the hydrogen peroxide system to achieve a biocidal effect equivalent to that of formaldehyde. Optimization may also involve increasing the quantity of hydrogen peroxide vapor introduced, extending the cycle time, or increasing the frequency and duration at which the cabinet fans pulse.

Only 1 virus, IBV, was used in the present study, and this may be interpreted as a limitation. However, the focus of the work was to provide a direct comparison between formaldehyde and hydrogen peroxide and to evaluate variables such as cabinet location and protein soiling in a single virus model. This approach could now be applied to screen multiple virus candidates to give process assurance when decontaminating MBSCs.

## Conclusions

Overall, the current volume of formaldehyde used for cabinet fumigation was more than enough to sufficiently reduce the titer of IBV. Formaldehyde has shown itself to be efficacious against IBV in environments containing high protein levels and is able to reach locations throughout a Class II MBSC. The hydrogen peroxide system was able to equal formaldehyde in achieving full IBV titer reduction within the cabinet workspace, but further optimization of the hydrogen peroxide system is required to achieve appropriate decontamination above the first HEPA filter of the cabinet. There is the question of whether full decontamination above the HEPA filter is required as the filter would be expected to trap virus particles. However, achieving kill after the HEPA filter gives assurance of decontamination throughout the matrix of the filter media. During servicing of the MBSC, it may be relevant to demonstrate adequate decontamination above the HEPA filter, but for fumigation, where simply cabinet workspace decontamination is desired, it could be argued to be not necessary. This study exclusively used the enveloped coronavirus IBV to quantify the fumigation efficacy, so the results could potentially be extrapolated to validate other enveloped viruses. However, nonenveloped viruses are known to generally be more environmentally resistant,^25,26^ and therefore the model developed in this article could be extended to include other viruses.
